# Case Report of Thrombosis of the Distal Aorta with Occlusion of Iliac Arteries in COVID-19 Infection

**DOI:** 10.5811/cpcem.2020.11.49593

**Published:** 2020-12-07

**Authors:** Andrew LaFree, Alexis Lenz, Christian Tomaszewski, Faith Quenzer

**Affiliations:** University of California, San Diego, Department of Emergency Medicine, San Diego, California

**Keywords:** SARS-CoV-2, COVID-19, arterial thromboembolism, coagulopathy, aortoiliac occlusion

## Abstract

**Introduction:**

The severe acute respiratory syndrome coronavirus 2 (SARS-CoV-2), which is responsible for the coronavirus disease of 2019 (COVID-19) pandemic, has been associated with a variety of prothrombotic sequelae. The pathogenesis of this hypercoagulability has not yet been fully elucidated, but it is thought to be multifactorial with overactivation of the complement pathways playing a central role. There is emerging evidence that the resulting complications are not confined to the venous circulation, and even in patients without typical respiratory symptoms or traditional risk factors, there is a significant rate of arterial thromboembolic disease in patients with SARS-CoV-2 infection.

**Case Report:**

We describe a patient presenting with bilateral leg pain without any respiratory symptoms or fever who ultimately was found to be COVID-19 positive and had thromboembolism of the aorta and bilateral iliac occlusion. This report reviews available evidence on the prevalence of arterial thromboembolism in COVID-19 patients and some proposed mechanisms of the pathophysiology of COVID-19-associated coagulopathy.

**Conclusion:**

It is important that the emergency physician maintain a high degree of suspicion for arterial thromboembolic disease in patients who are infected with COVID-19 even in the absence of typical respiratory symptoms. Additionally, COVID-19 should be considered in patients with unexplained thromboembolic disease, as this may increase the detection of COVID-19.

## INTRODUCTION

The severe acute respiratory syndrome coronavirus 2 (SARS-CoV-2) may create numerous complications outside of the respiratory tract. Coagulopathies have also been observed as complications associated with the virus.^1^–^3^ These prothrombotic sequelae are not confined to the venous circulation. Here we present a case of a patient with thrombosis of the distal abdominal aorta and occlusion of bilateral iliac arteries complicated by severe rhabdomyolysis who presented with lower extremity pain and weakness in the setting of a coronavirus disease 2019 (COVID-19) infection. Although cases of lower extremity arterial thrombosis have been described before,^1^–^3^ our case is unique in that the patient was relatively young, presented without respiratory symptoms, and had bilateral leg involvement necessitating amputations. This case demonstrates the importance of maintaining a high index of suspicion for arterial thrombosis in patients with COVID-19 infection even in the absence of typical respiratory symptoms or other evidence of severe disease. It also important within this context to also consider COVID-19 in unexplained thrombosis.

## CASE REPORT

A 57-year-old male with a history of type 2 diabetes and hypertension presented to the emergency department (ED) with low back pain radiating into his legs worsening over the prior three days. The patient was not able to identify any clear precipitating factors or trauma. He described the pain as moderate in intensity and of a “burning” quality. Prior to being seen in our ED the patient was seen at an outside clinic in Mexico where he was given a “Toradol shot and steroids” and diagnosed with sciatica. The patient denied any other symptoms including fever, cough, shortness of breath, chills, or weakness.

The patient’s vital signs on presentation were normal except for a heart rate of 101 beats per minute. He was noted to have a blood pressure of 119/79 millimeters of mercury (mm Hg), a pulse oxygen saturation of 96%, and a temperature of 98.2° Fahrenheit (F). The patient’s exam was unremarkable except for pain to palpation of bilateral lower extremities. He was not noted to have any weakness or neurological deficit and had normal bilateral dorsalis pedis pulses. The patient had a blood glucose within normal limits. He was given pain medication and steroids and felt improvement of his symptoms. As the pain seemed to improve, his symptoms were attributed to sciatica and he was discharged home.

Two days later the patient returned to the ED reporting worsening pain in his flank and legs and bilateral lower extremity weakness. He reported moderate to severe pain with movement, which had limited his ability to ambulate. The patient complained of some dysuria but continued to deny other symptoms including fever, cough, shortness of breath, chest pain, nausea, vomiting, diarrhea, numbness or inability to urinate. At this presentation, the patient had a heart rate of 66 beats per minute, blood pressure was 120/79 mm Hg, oxygen saturation was 95% on room air, and his temperature was 97.4°F. He reported that his pain on arrival was 10/10. He was found to have full range of motion, normal sensation, and equal dorsalis pedis pulses bilaterally in his lower extremities. He was noted to have pain with movement of his lower extremities, and endorsed tingling. He had normal rectal tone. His lower extremities were noted to be hyporeflexic.

The patient was given pain medication and his laboratory results revealed an elevated white blood cell count of 23.5 × 10^9^/liter (L) (reference range 4.0–10.0 × 10^9^/L) with normal hemoglobin, hematocrit, and platelets. His comprehensive metabolic panel was normal with the exception of bicarbonate of 22 milliequivalents (mEq)/L (23–30 mEq/L), an elevated blood urea nitrogen of 60 milligrams per deciliter (mg/dL) (7–20 mg/dL), an alanine aminotransferase of 242 units (U)/L (7–56 U/L), and an aspartate aminotransferase of 617 U/L (normal 0–35 U/L). His creatine phosphokinase was markedly elevated at 26,818 U/L (20–600 U/L). His D-dimer was 562 nanograms per milliliter (ng/mL) (< 250 ng/mL). His lactate dehydrogenase was 2601 U/L (140–280 U/L). The urinalysis demonstrated 2+ blood and 30 red blood cells. Based on his urinalysis, a computed tomography (CT) abdomen and pelvis was also ordered to evaluate for nephrolithiasis.

**CPC-EM Capsule**

What do we already know about this clinical entity?

Severe acute respiratory syndrome coronavirus 2 infection often presents as respiratory illness and hypoxia, but has been associated with significant coagulopathy.

What makes this presentation of disease reportable?

We present the case of a large aortoiliac occlusion in a coronavirus disease of 2019 (COVID-19) patient with no initial respiratory complaints.

What is the major learning point?

Major coagulopathic complications such as stroke, pulmonary embolism, coronary artery embolism, and arterial thrombosis have been observed in COVID-19 patients.

How might this improve emergency medicine practice?

Providers should consider arterial thrombosis in COVID-19 patients with neurovascular compromise and coagulopathy. Prompt imaging can improve morbidity and mortality.

The CT of his abdomen did not demonstrate any significant intra-abdominal abnormalities, but he was noted to have diffuse patchy infiltrates in his lower lungs. A chest radiograph demonstrated patchy diffuse bilateral infiltrates. These findings prompted COVID-19 testing, which resulted as positive despite lack of any upper respiratory symptoms. A lumbar spine CT was completed, which was also unremarkable. While in the ED, his pain and weakness worsened and ultimately a magnetic resonance imaging (MRI) of his lumbar spine and a venous duplex of his lower extremities were also obtained and he was admitted for pain control and further workup. The MRI was unremarkable and his lower extremity venous duplex ultrasonography did not demonstrate any acute deep vein thrombosis. Within two hours of his ED stay, an arterial duplex of his lower extremities was performed and demonstrated no blood flow in the right dorsalis pedis artery or left posterior tibial, anterior tibial, or dorsalis pedis arteries. A computed tomographic angiography (CTA) of the aorta and lower extremities demonstrated diffuse arterial insufficiency with thrombosis of distal abdominal aorta and occlusion of bilateral iliac arteries (Image). He was then placed on a heparin drip. The patient’s respiratory status deteriorated over the next few days and he required endotracheal intubation for acute respiratory failure. He was transferred to an outside hospital for possible thrombectomy.

The thrombectomy was successful; however, the patient’s lower extremities were not salvageable and he required bilateral above knee amputations. His hematology workup revealed slight decrease in protein S 39% (reference range 66–143%) with normal protein C, complement, antithrombin 3, homocysteine, and cardiolipin antibodies, and no evidence of disseminated intravascular coagulation (DIC). Ultimately, the patient’s respiratory status improved and he was extubated. At the time of writing he remained hospitalized on enoxaparin with discharge planning ongoing.

## DISCUSSION

There is ample documentation of the relatively high rate of thromboembolic complications in critically ill COVID-19 patients. Prior studies of thrombotic events have been estimated to occur in 3.7–45% of COVID-19 cases.^2^,^5^ Prophylaxis and management of thromboembolic complications is an important component of COVID-19 therapy. One retrospective study of 449 admitted severe COVID-19 patients showed that the administration of prophylactic heparin was associated with a marked reduction in mortality (40% vs 64%).^6^ However, other observational studies have shown less consistent benefit.^6^,^7^

As in our case, there are reports that demonstrate an association of lower extremity arterial thromboses such as aortoiliac thromboses and SARS-CoV-2 infection. Vulliamy and colleagues published two cases of patients with COVID-19 pneumonia who also presented with acute thrombotic occlusion of the descending aorta.^3^ A recent case series of 16 patients showed a high incidence of aortic and lower extremity arterial thromboses in patients with COVID-19 infection.^1^ Prominent features include ischemic leg symptoms, large thrombus burden, and involvement of proximal vessels. Many of these patients required amputation (25%) or had complications that resulted in death (38%). Another cohort of acute lower extremity ischemia in patients diagnosed with COVID-19 pneumonia had an overall mortality rate of 40%.^8^ A summary of these case reports and case series is found in the table.

Patients with COVID-19 infection can have pathology consistent with systemic hypercoagulable state, marked by microvascular thrombotic disorders and elevated D-dimer.^9^ It can occur despite antithrombotic prophylaxis and be refractory to full anticoagulation.^10^ In our case anti-phospholipid antibodies were not elevated, consistent with what has been seen in other series of COVID-19 patients with thrombotic complications.^11^ Our patient also did not have evidence of DIC. The modest protein S deficiency manifested by the patient has been hypothesized to contribute to cytokine storm in COVID-19 and could be the result of exuberant dysregulated blood coagulation.^12^

The pathogenesis of hypercoagulability associated with COVID-19 has yet to be fully elucidated, but it is thought to be multifactorial with contributions from excessive inflammation, hypoxia, immobilization and DIC. Additionally, complement pathways may play a role in the pathogenesis of this hypercoagulability. Deposition of complement components has been shown to cause endothelial cell injury and subsequently activate the clotting pathway and lead to fibrin deposition.^13^ Multiple studies have documented that in at least a subset of COVID-19 patients, respiratory failure is the result of sustained, complement-mediated thrombotic microvascular injury and activation of catastrophic positive feedback loops with the coagulation system.^9^

Moreover, viral interaction with angiotensin converting enzyme (ACE2) receptors has been postulated to cause an overactivation of complement systems. Research has shown that SARS-CoV-2 enters cells through ACE2 receptors, which leads to downstream effects of increased angiotensin II. Angiotensin II is associated with inflammation and fibrosis and the resulting increases of oxidative stress and complement activation.^13^ Patients with severe COVID-19 are observed to have excessive complement activation with elevated lactate dehydrogenase, D-dimer, bilirubin, anemia, and decreased platelets all potentially leading to thrombotic microangiopathy.^10^,^12^,^13^ Therefore, current research has been targeting complement inhibition for treatment of COVID-19 systemic thrombosis.^7^,^14^

## LIMITATIONS

Literature regarding the association between SARS-CoV-2 and thromboembolism is still rapidly evolving. A recent, large, retrospective study showed that the odds of pulmonary embolism in patients who are diagnosed with COVID-19 vs non-COVID-19 infected patients were not significantly different between the two groups.^15^ This may shed some doubt on the association of SARS-CoV-2.

We also could not establish strong causal effect of coagulopathic state with the outcome of the large aortoiliac occlusion in this particular patient. The patient did not initially report a personal or family history of coagulopathies. Additionally, we do not know whether the patient had primary, underlying protein S deficiency, thus making him more susceptible to an increasingly prothrombic state. A hypercoagulable state induced by concomitant COVID-19 infection may have increased the potential for thrombosis, which could still have made SARS-CoV-2 infection a significant contributing factor. Without known pre-existing risk factors for thromboembolism, the patient still had a devasting thrombotic event. In the presence of abnormal vital signs, neurovascular compromise and coagulopathy, emergency physicians should maintain a high suspicion for thromboembolic events especially if the patient has concomitant COVID-19.

## CONCLUSION

We report a case of a patient with diffuse lower aortic and bilateral iliac arterial thrombosis who tested positively for COVID-19 while lacking any of the common early respiratory complaints. Early recognition of this complication from COVID-19 may have improved outcome, although the literature is unclear on the benefit of anticoagulation. Regardless, emergency physicians should prompt expedient imaging given presence of leg pain and ischemia in a patient with SARS-CoV-2. Further workup is indicated and early thrombectomy can aid in improved morbidity and mortality.

## Figures and Tables

**Image:**
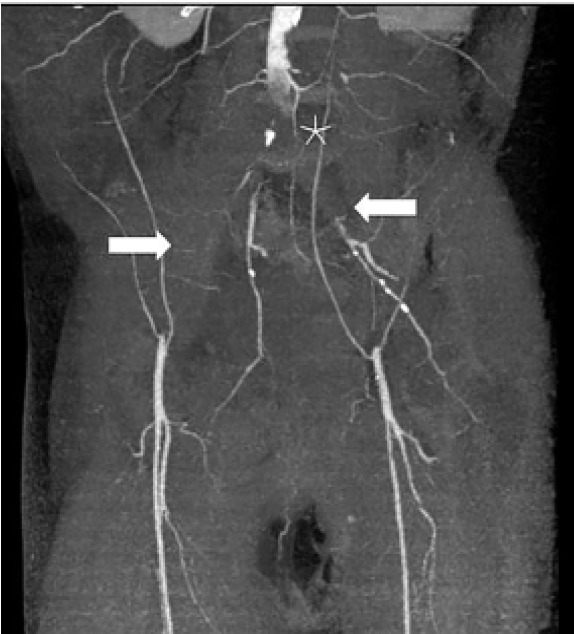
Computed tomographic angiography of abdomen and lower extremities with extensive thrombosis of distal abdominal aorta (asterisk) and occlusion of bilateral iliac arteries (arrows).

**Table:** Characteristics of selected case studies demonstrating presence of arterial thromboembolism in coronavirus disease of 2019 positive patients.

Author/Year	Age	Gender	Co-morbidities	Associated pneumonia	Location of arterial thrombus	Thrombectomy	Amputation or surgical intervention	Death
Veyre (2020)^3^	24	M	None	N	Common femoral artery	Y	N	N
Vulliamy (2020)^4^	60	M	None	Y	Infra-renal aorta & bilateral common iliac arteries	Y	N	N
Levolger (2020)^2^	75	M	None	Y	Descending thoracic aorta & superior mesenteric artery	N (catheter directed thrombolysis)	Small bowel resection	N
	50	M	None	Y	Right common Iliac artery	Y (received thrombolysis)	N	N
	55	M	None	N	Subclavian artery occlusion	N	N	N
	62	M	Unknown	N	M1 occlusion with subtotal internal carotid stenosis	Y	N	N

Goldman (2020)^1^	Mean Age	Gender	Co-Morbidities	Symptoms	Presence of thrombus		Death or Amputation	

Retrospective propensity score-matched study comparing 16 COVID-19+ patients receiving lower extremity CTA to Control Group	70	7 F 9 M	Variable HTN: 13 DM: 8 HLD:8 PVD: 8	5 patients presented with only leg symptoms; 11 patients had additional systemic symptoms	All COVID-19+ patients had at least one thrombus between aorta and distal lower extremity arteries		10/16 patients ultimately progressed to death or amputation	

*Y,* yes; *N,* no; *F,* female; *M,* male; *HTN,* hypertension; *DM,* diabetes; *HLD,* hyperlipidemia; *PVD,* peripheral vascular disease; *CTA,* computed tomographic angiography; *M1,* M1 segment as it originates at the carotid bifurcation and terminates as the middle cerebral artery.
